# Clinical characteristics of severe influenza virus-associated pneumonia complicated with bacterial infection in children: a retrospective analysis

**DOI:** 10.1186/s12879-023-08536-x

**Published:** 2023-08-21

**Authors:** Qiong-yu Wang, Lin Yuan, Jia-yi Lin, Zhi-qiang Zhuo, Yong-mei Wang, Si-si Li, Min Zhang, Xing-dong Wu

**Affiliations:** 1 Xiamen Children’s Hospital/Children’s Hospital of Fudan University at Xiamen, Xiamen, 361006 Fujian China; 2 University of Manchester, Oxford Road, Manchester, M13 9PL UK

**Keywords:** Bacteria, Children, Infection, Influenza virus, Pneumonia

## Abstract

**Background:**

We aimed to investigate the clinical characteristics of severe influenza virus-associated pneumonia complicated with bacterial infection in children.

**Methods:**

We retrospectively analysed data concerning 64 paediatric patients with severe influenza virus-associated pneumonia who had been treated at our hospital. The patients were divided into observation (44 patients) and control (20 patients) groups, based on the presence or absence of concomitant bacterial infection, and clinical data were compared between the groups.

**Results:**

The mean age in the observation group was 2.71 ± 1.44 years, 42 (95.45%) were aged ≤ 5 years, and 18 (40.9%) had underlying diseases. The mean age in the control group was 4.05 ± 2.21 years, 13 (65%) were aged ≤ 5 years, and 3 (15%) had underlying diseases. There was a statistically significant difference in patient age and the proportion of patients with underlying diseases (*P* < 0.05). The observation group had higher duration of fever values, a higher number of patients with duration of fever ≥ 7 days, a higher incidence of gasping, and a higher incidence of seizures/consciousness disturbance, and the differences were statistically significant (*P* < 0.05). Secondary bacterial infections in the observation group were mainly due to gram-negative bacteria, with *Haemophilus influenzae* and *Moraxella catarrhalis* being the most common pathogens. The observation group had a higher proportion of patients treated in the paediatric intensive care unit and a longer hospital stay, and the differences were statistically significant (*P* < 0.05).

**Conclusion:**

Severe influenza virus-associated pneumonia complicated with bacterial infection was more common in children aged ≤ 5 years. Younger patients with underlying diseases were more susceptible to bacterial infection (mainly due to gram-negative bacteria). The timely administration of neuraminidase inhibitors and antibiotics against susceptible bacteria is likely to help improve cure rates.

Influenza, also known as the ‘flu, causes periodic pandemics. It has been estimated that seasonal influenza affects approximately 1 billion people worldwide every year. Among paediatric populations, the influenza infection rate has been reported to be as high as 20–30% [1, 2], which is considerably higher than that in adults. Pneumonia is a common cause of hospitalisation in patients infected with the influenza virus, and infected children have a high tendency to develop severe influenza virus-associated pneumonia, which may be life-threatening [3, 4]. Previous studies have found that patients infected with the influenza virus are prone to developing secondary bacterial infection, which usually occurs within 7 days of influenza virus infection and may aggravate disease severity and affect the prognoses of such patients [5]. This issue is particularly prominent in the paediatric influenza pneumonia population. Given the current lack of studies on severe influenza virus-associated pneumonia complicated with bacterial infection in children in China, we aimed to analyse the clinical characteristics of paediatric patients with concomitant severe influenza virus-associated pneumonia and bacterial infection who had been treated at Xiamen Children’s Hospital, to enhance understanding of this disease and help determine appropriate disease prevention strategies and treatment for clinicians.

## Patients and methods

### Patients

In this retrospective study, we analysed data concerning hospitalised patients with severe influenza virus-associated pneumonia at Xiamen Children’s Hospital between January 1, 2018 and December 31, 2021. We divided the patients into two groups based on the presence or absence of concomitant bacterial infection. Patients with concomitant bacterial infection were included in an observation group, while those without were assigned to a control group. A diagnosis of severe pneumonia was made if any of the following criteria were met: (i) a poor general condition; (ii) consciousness disturbance; (iii) cyanosis; (iv) rapid breathing (age < 2 months: respiratory rate (RR) ≥ 60 breaths/min; age ≥ 2 months to 1 year: RR ≥ 50 breaths/min; age > 1–5 years: RR ≥ 40 breaths/min; age > 5 years: RR ≥ 30 breaths/min); (v) dyspnoea (moaning, nasal flaring, and the “three depression” sign, i.e., depression of the suprasternal fossa, supraclavicular fossa, and intercostal space); (vi) pulse oxygen saturation ≤ 92%; (vii) refusal to eat or signs of dehydration; (viii) plain chest radiography or computed tomography (CT) scan showing lung infiltration, pneumothorax, lung necrosis, and lung abscess in ≥ two-thirds of one lung, and; (ix) extrapulmonary complications.

Inclusion criteria concerning the observation group were as follows: (i) meeting the diagnostic criteria for influenza: presence of epidemiological history, influenza-like symptoms, and at least one positive result for influenza antigen or nucleic acid tests; (ii) meeting the diagnostic criteria for severe pneumonia; (iii) patient age: 1 month–12 years; (iv) clinical manifestations of infection that could not be explained by influenza, namely, elevated leukocytes, C-reactive protein, and procalcitonin during anti-infective treatment; positive blood cultures, positive cerebrospinal fluid, pleural effusion, sputum, and alveolar lavage fluid, with the colony requirements for positive sputum culture being as follows: ≥10^5^ CFU/ml in tracheal aspirate, ≥10^4^ CFU/ml in alveolar lavage fluid; and the presence of bacteria and leukocyte infiltration and phagocytosis under sputum smear microscopy.

Exclusion criteria were as follows: (i) incomplete data for statistical analysis; (ii) specimens with contamination or bacterial colonisation; sputum specimens with > 10 squamous epithelial cells/high power field (HPF), and; (iii) < 25 leukocytes/HPF, which were considered unfit for use. If pathogens were isolated from non-sterile sites without imaging or clinical signs of infection, bacterial colonisation was considered present. Other cases of severe influenza virus-associated pneumonia that had not been included in the observation group were included in the control group. The present study was approved by the ethics committee of our hospital (Approval number: XCSER [2022] 38).

### Methods

#### Data collection

Data concerning patients with severe influenza virus-associated pneumonia who had been treated at our hospital between January 1, 2018 and December 31, 2021 were retrieved from the electronic inpatient records system of our hospital. Baseline information in relation to clinical data, symptoms, signs, auxiliary examination results, treatment details, prognoses, and patient outcomes were collected.

#### Pathological examination methods

Influenza antigen testing was performed using the colloidal gold assay (Standard Diagnostics Inc., South Korea), bacteria were detected using a fully automated microbial mass spectrometer (Microflex LF/SH, Shanghai), and bacterial susceptibility to antibiotics was tested using a fully automated antimicrobial susceptibility testing system (VITEK 2 Compact, France). All methods were carried out accordance with relevant guidelines.

#### Statistical analysis

Data were statistically analysed using SPSS version 23.0 software. Normally distributed measurement data were expressed as mean ± standard deviation (SD) and compared between groups using a *t*-test. Non-normally distributed measurement data were expressed as median (interquartile range) [M (P_25–_P_75_)] and compared between groups using a Mann-Whitney U test. Count data were expressed as the number (%) of patients and compared between groups using a *χ*^2^ test.

## Results

### General data

In total, 64 patients with severe influenza virus-associated pneumonia were included in this study. The observation group comprised 44 paediatric patients (boys, *n* = 32; girls, *n* = 12). The mean age was 2.71 ± 1.44 years, with 15 (34.1%) patients aged ≤ 2 years, 27 (61.4%) aged 2–5 years, and 2 (4.5%) aged > 5 years. Within the observation group, 35 (79.6%) patients had influenza type A infection and 9 (20.4%) had influenza type B infection. Concomitant infections included *Haemophilus influenzae* (*H. influenzae)* infection (*n* = 18 patients), *Moraxella catarrhalis* (*M. catarrhalis*) infection (*n* = 12 patients), *Pseudomonas aeruginosa* infection (*n* = 3 patients), *Staphylococcus aureus* infection (*n* = 4 patients), *Streptococcus pneumoniae* infection (*n* = 6 patients), and infection due to ≥ 2 types of bacteria in 1 patient. Eighteen (40.9%) patients had underlying diseases, which included febrile seizures (*n* = 8), congenital heart disease (*n* = 4), hydrocephalus (*n* = 1), developmental disorder (*n* = 1), epilepsy (*n* = 2), and asthma (*n* = 2).

The control group comprised 20 paediatric patients (boys, *n* = 13; girls, *n* = 7). The mean age was 4.05 ± 2.21 years, with 6 (30%) patients aged ≤ 2 years, 7 (35%) aged 2–5 years, and 7 (35%) aged > 5 years. Within the control group, 15 (75%) patients had influenza type A infection, 5 (25%) had influenza type B infection, and 3 (15%) had underlying diseases, including febrile seizures, congenital heart disease, and developmental disorder (1 patient each, respectively). Table 1 shows a comparison of general data between the observation and control groups.

### Clinical characteristics

Among the observation group patients, 43 developed pyrexia with a peak temperature of 39.8 °C (range, 39.5–40.08 °C) and a pyrexia duration of 6 (5.25–7) days, and 21 (47.73%) patients had a pyrexia duration of ≥ 7 days. Other clinical symptoms included cough in all patients, gasping in 31 (70.45%), seizures/consciousness disturbance in 26 (59.09%), and audible pulmonary rales in 36 (81.82%) patients. In 23 (52.27%) patients, pulmonary imaging results suggested the presence of pulmonary consolidation and atelectasis. Moreover, 2 patients had respiratory failure, 3 had septic shock, 2 had toxic encephalopathy, 3 had liver injury, 1 had metabolic acidosis, and 1 patient had tracheal stenosis.

In the control group, all 20 patients developed pyrexia with a peak temperature of 39.3 °C (range, 39–40.2 °C) and a pyrexia duration of 5 (3–6) days, and 4 (20%) patients had a pyrexia duration of ≥ 7 days. Other clinical symptoms included cough in all patients, gasping in 8 (40%), seizures/consciousness disturbance in 5 (25%), and audible pulmonary rales in 15 (75%) patients. In 10 (50%) patients, pulmonary imaging results suggested the presence of pulmonary consolidation and atelectasis. There was also 1 patient with respiratory failure, 1 in septic shock, 2 with brainstem encephalitis, 1 with liver injury, 1 with myocardial damage, and 2 with agranulocytosis. Table 2 shows a comparison of clinical characteristics between the observation and control groups.

### Bacterial culture and susceptibility to antibiotics

A total of 46 positive culture specimens were collected from the 44 patients in the observation group, of which 44 were oronasal sputum specimens and 2 were alveolar lavage fluid specimens. In 2 patients, positive cultures were detected in both the oronasal sputum and alveolar lavage fluid specimens. All *H. influenzae* cultures were susceptible to ceftriaxone, cefotaxime sodium, and meropenem, showing resistance rates of 61.7%, 76.6%, 27.7%, and 6.4% to ampicillin, co-trimoxazole, cefuroxime, and ampicillin/sulbactam, respectively. All *M. catarrhalis* cultures were susceptible to ampicillin/sulbactam, amoxicillin/sulbactam, and ciprofloxacin, and exhibited resistance rates of 59.4% and 13.3% to ampicillin and co-trimoxazole, respectively.

### Treatment and outcomes

The observation group patients were treated with neuraminidase inhibitors (NAIs), with 36 patients having received oseltamivir via oral administration/nasal feeding, 6 patients having received peramivir via intravenous infusion, and 2 patients having received oral oseltamivir followed by peramivir via intravenous infusion. In 31 patients, NAIs were used for the first time at > 48 h after disease onset. Antibiotics were administered to all patients over a duration of 7 (range, 5–8) days. Eighteen (40.9%) patients underwent fibreoptic bronchoalveolar lavage therapy and 26 (59.1%) were treated in the paediatric intensive care unit (PICU). The average length of hospital stay in the observation group was 12 (range, 9–14) days. One patient aged ≤ 5 years who had underlying epilepsy died of haemophagocytic lymphohistiocytosis.

The control group patients were also treated with NAIs, with 19 patients receiving oseltamivir via oral administration/nasal feeding and 1 patient receiving oral oseltamivir followed by peramivir via intravenous infusion. In 12 patients, NAIs were used for the first time at > 48 h after disease onset. Six (30%) patients underwent fibreoptic bronchoalveolar lavage therapy and 5 (25%) were treated in the PICU. The control group had an average length of hospital stay of 7 (range, 6–12.8) days and no patients died. Table 3 shows a comparison of treatment and outcomes between the observation and control groups.

**Table 1:** A comparison of general data between the observation and control groups

	Observation group (*n* = 44)	Control group (*n* = 20)	*P*-value
Sex (male), no. (%)	32 (72.7)	13 (65)	0.531
Age (mean ± SD, years)	2.71 ± 1.442	4.05 ± 2.21	0.005
Age ≤ 5 years, no. (%)	42 (95.5)	13 (65)	0.001
Presence of underlying diseases, no. (%)	18 (40.9)	3 (15)	0.041
Influenza type (type A), no. (%)	35 (79.6)	15 (75)	0.683

*no.(%)* number(percent), *SD* standard deviation

**Table 2:** A comparison of clinical characteristics between the observation and control groups

	Observation group (*n* = 44)	Control group (*n* = 20)	*P*-value
Pre-hospitalisation duration of fever [^a^, days]	4 (2–6)	1 (1–5.8)	0.218
Peak temperature of pyrexia [^a^, °C]	39.8 (39.5–40.1)	39.3 (39–40.2)	0.694
Pyrexia duration [^a^, days]	6 (5.3, 7)	5 (3, 6)	0.006
Pyrexia duration ≥ 7 days, no. (%)	20 (45.5)	4 (20)	0.035
Gasping, no. (%)	31 (70.5)	8 (40)	0.021
Seizures/consciousness disturbance, no. (%)	26 (59.1)	5 (25)	0.011
Pulmonary rales, no. (%)	36 (81.8)	15 (75)	0.53
WBC [^a^x10^9^, (count/l)]	7.1 (5.7–9)	7.6 (4.4–10)	0.857
NEU [^a^x10^9^, (count/l)]	4 (3.1, 5.6)	4.6 (2.3, 7.6)	0.775
LY [^a^x10^9^, (count/l)]	2.07 (1.57–3.20)	1.78 (0.79–3)	0.150
MO [^a^x10^9^, (count/l)]	0.78 (0.56–1.23)	0.69 (0.37–1.17)	0.153
NEU% (mean ± SD,%)	56.54 ± 15.94	61.44 ± 19.11	0.431
LY% [^a^,%]	30.2 (20.4–40.9)	25.3 (14–40.9)	0.402
MO% (mean ± SD,%)	10.95 ± 4.33	9.04 ± 4.07	0.624
PLT [^a^x10^9^, (count/l)]	329.5 (264–445)	217 (153–320)	0.001
CRP [^a^, mg/l]	35.5 (15.1–59.3)	5.54 (2.7–37)	0.02
PCT [^a^, ng/ml]	1.89 (1.03–2.31)	0.39 (0.12–0.57)	0.006
Other viral infections, no. (%)	5 (11.4)	0 (0)	0.375
Concomitant MP infection, no. (%)	6 (13.6)	7 (35)	0.068
CK [^a^, u/l]	148 (100–216)	145 (103–190)	0.95
CK-MB [^a^, u/l]	24 (20–31)	26.5 (18.8–30.5)	0.862
LDH (mean ± SD, u/l)	423.7 ± 130.4	354.17 ± 106.3	0.036

^a^ *M(P*_*25*_*-P*_*75*_*)* nterquartile range, *no.(%)* number (percent), *SD* standard deviation, *WBC* white blood cell, *NEU* neutrophil, *LY* lymphocyte, *MO* monocyte, *PLT* platelet, *CRP* C-reactive protein, *PCT *procalcitonin, *MP* mycoplasma, *CK* creatine kinase, *CK-MB* creatine kinase MB lsoenzyme, *LDH* lactate- dehydrogenase

**Table 3:** Comparison of treatment between the observation and control groups

	Observation group (*n* = 44)	Control group (*n* = 20)	*P-*value
Bronchoscopic treatment, No. (%)	18 (40.9)	6 (30)	0.316
Treatment in PICU, No. (%)	26 (59.1)	5 (25)	0.011
Length of hospital stay [M (P_25_–P_75_), days]	12 (9, 14)	7 (6, 12.8)	0.032
Length of hospital stay ≥ 7 days, No. (%)	34 (77.3)	12 (60)	0.154

*M(P*_*25*_*-P*_*75*_*)* nterquartile range, *no.(%)* number (percent), *PICU* paediatric intensive care unit

## Discussion

Pneumonia due to influenza virus infection is a common cause of hospitalisation among children. In severe cases, patients may experience respiratory failure, septic shock, or even life-threatening conditions [3, 4, 6]. One study reported that > 10,000 children around the world aged ≤ 5 years die from influenza virus-associated pneumonia annually, with most being paediatric patients with influenza virus-associated pneumonia complicated with bacterial infection [7]. Therefore, clinicians should pay greater attention to concomitant influenza virus-associated pneumonia and bacterial infection in children, particularly in children with severe illness. In our study, the average age of the observation group was significantly lower than that of the control group, indicating that younger age is associated with a higher risk of secondary bacterial infection in severe influenza virus-associated pneumonia. We also found that children aged ≤ 5 years comprised 95.5% (42/44) of the patients in the observation group, which was similar to the findings reported by Zhou et al. [1]. Besides, the observation group had a higher proportion of patients with underlying diseases than the control group. There was also one death in the observation group, which occurred in a patient with a history of epilepsy.

Given these findings, the possibility of concomitant bacterial infection in patients aged ≤ 5 years with severe influenza virus-associated pneumonia should be taken seriously. Relevant guidelines developed by experts in China and other countries have stated that children aged ≤ 5 years are not only susceptible to influenza virus infection but are also a high-risk population for secondary bacterial infection [8, 9]. This susceptibility may be explained partly by the fact that influenza virus replication in the respiratory tract reduces ciliary beat frequency in the airway, and the haemagglutinin and neuraminidase proteins of the virus alter the surface protein receptors of infected cells, thereby providing binding sites for bacterial adhesion [10]. Another key cause is immune cell dysfunction induced by the influenza virus or host factors. Previous research has indicated that immune cells such as alveolar macrophages, neutrophils, and natural killer cells form the second line of defence of the human body against bacterial invasion. However, influenza virus infection can destroy this line of defence, resulting in the weakening of phagocytosis, chemotaxis, and intracellular killing; such a mechanism is especially prominent in infected patients with reduced immune function [11]. In addition, these findings also suggest that children with underlying diseases may be more prone to developing concomitant bacterial infection when they have severe influenza, and the presence of underlying diseases is a risk factor for progression to severe disease or even death in paediatric patients with influenza [12]. Therefore, particular attention should be paid to paediatric patients with influenza and underlying diseases in clinical practice.

The reported incidence rates of influenza virus infection complicated with bacterial infection have varied over recent years. In a 2009 multicentre study conducted at 17 hospitals in China, 14.0% of children with influenza virus infection had a concomitant bacterial infection [13]. A 2018 study involving 838 paediatric patients with influenza in 35 PICUs in the United States reported the presence of concomitant bacterial infection in 274 (32.7%) patients, [14] a higher incidence rate than that reported in the 2009 study. Our results showed that the proportion of patients with influenza virus infection and with concomitant bacterial infection was 68.74% (44/64), which was higher than the aforementioned incidence rates. Certain biases may exist in our study due to its single-centre design and small sample size; however, the overall increasing trend in the proportion of paediatric patients with severe influenza virus-associated pneumonia complicated with bacterial infection cannot be ignored, especially in severe paediatric cases. We also observed clear changes in the bacterial spectra of patients with concomitant influenza and bacterial infection. The previously mentioned multicentre study concerning patients with influenza and bacterial coinfection in China found that gram-positive bacteria accounted for > 60% of all cases [13]. The results of a meta-analysis of > 3000 cases of influenza and bacterial coinfection in various countries in 2003–2014 also showed that gram-positive bacteria predominated, with *Streptococcus pneumoniae* and *Staphylococcus aureus* infections being the most common [15]. Our results differed from those of the aforementioned studies in that gram-negative bacterial infections accounted for 75% (33/44) of patients with severe influenza virus-associated pneumonia complicated with bacterial infection, with *H. influenzae* and *M. catarrhalis* accounting for 40.9% (18/44) and 27.3% (12/44) of patients, respectively. Possible reasons for the predominance of gram-negative bacteria in the present study are as follows. First, in recent years, changes have occurred in the bacterial spectra of lower respiratory tract infections in children, causing the incidence rate of gram-negative bacterial infections to exceed that of gram-positive bacterial infections. A 2015 Chinese study by Li et al. [16] reported that gram-negative bacteria accounted for approximately 74.1% of lower respiratory tract infections in children, which was significantly higher than the incidence rate for gram-positive bacterial infections. In 2019, Lim et al. [17] reported that, in Korea, *H. influenzae* accounted for 25.6% of cases of community-acquired pneumonia with viral and bacterial coinfection and that the rate of coinfection exhibited an increasing trend, which is consistent with the *H. influenzae* infection rate of 40.9% (18/44) observed in our study. Second, differences in bacterial strains causing secondary bacterial infections may exist between patients with severe influenza virus-associated pneumonia and those with common influenza virus infection. Kim et al. [18] reported that secondary bacterial infections in mechanically ventilated patients with influenza type A infection were most commonly due to *Acinetobacter baumannii*, *Klebsiella pneumoniae*, and *Pseudomonas aeruginosa*, which are all gram-negative bacteria. Third, factors such as differences in regional climate and sample size may also affect the conclusions obtained in different studies. Nonetheless, paying attention to changes in the bacterial spectra of concomitant bacterial infection in paediatric influenza virus-associated pneumonia is of practical significance for early diagnosis confirmation and timely administration of targeted antibacterial treatment.

Influenza and bacterial coinfection lack specific manifestations in the early stage and can only be confirmed through a combined analysis of clinical symptoms, aetiology, and other laboratory examination results. Our findings showed that patients in the observation group were more prone to longer pyrexia duration and clinical symptoms such as gasping, seizures, and consciousness disturbance than patients in the control group. This suggests that the development of the aforementioned symptoms in children with severe influenza virus-associated pneumonia may serve as a warning sign of the possibility of concomitant bacterial infection. The observation group also exhibited significant increases in inflammatory indicators such as C-reactive protein and procalcitonin levels, indicating that these elevated levels in paediatric patients with severe influenza virus-associated pneumonia may assist in the early identification of bacterial infection. In addition, lactate dehydrogenase (LDH) levels were also significantly elevated in the observation group compared with the control group. A retrospective study of Japanese patients with pneumonia showed that the LDH level can serve as an indicator of lung tissue damage in children [19]. Our study results also suggest that LDH may also be used as a predictive indicator of the presence or absence of concomitant bacterial infection in children with severe influenza virus-associated pneumonia. However, further deliberation is required for the establishment of specific quantitative criteria.

In this study, the bacterial species most commonly detected in the patients were *H. influenzae* and *M. catarrhalis*. We found that *H. influenzae* was susceptible to cefotaxime, ceftriaxone, and meropenem but exhibited a resistance rate of 61.7% to ampicillin, which is close to the resistance rate of 69.19% reported by Mai et al. [20]. This result suggests that ampicillin may no longer be considered the drug of choice for the treatment of *H. influenzae* infection. Third-generation cephalosporins may instead be selected for initial empiric treatment. *M. catarrhalis* was susceptible to ampicillin/sulbactam, amoxicillin/clavulanate potassium, and ciprofloxacin, and exhibited resistance rates of 59.4% and 13.3% to ampicillin and co-trimoxazole, respectively. Therefore, we recommend the use of penicillin-based compound formulations as first-choice drugs for initial empiric treatment of *M. catarrhalis* infection in children. Ricketson et al. [21] reported that patients who did not receive standardised antibiotic treatment had a 5.6-fold increase in case fatality rates compared with a standardised treatment group. Similarly, we found that satisfactory therapeutic effects could be achieved with early administration of antibiotics against susceptible bacteria in the observation group.

This study had some limitations. First, this was a single-centre retrospective study that included patients from the past four years. Therefore, the data used for analysis were limited and may not fully reflect the characteristics of severe influenza virus-associated pneumonia complicated with bacterial infection in children. Second, all patients included in this study originated from Xiamen, Fujian Province, in Southern China, which may have created certain biases given the existence of genetic variation and differences in prevalent viral and bacterial strains among different geographical regions. In future research, we aim to address these shortcomings and increase the sample size to enhance the accuracy of our results.

In conclusion, severe influenza virus-associated pneumonia complicated with bacterial infection was found to be more common in paediatric patients aged ≤ 5 years. Younger patients with underlying diseases were more susceptible to bacterial infection. Secondary bacterial infections were mainly due to gram-negative bacteria, with *H. influenzae* and *M. catarrhalis* being the most commonly detected pathogens in the patients. The clinical manifestations of severe influenza virus-associated pneumonia complicated with bacterial infection lacked specificity. However, the following may serve as warning signs of the possibility of concomitant bacterial infection: severe clinical manifestations such as persistent high-grade pyrexia, consciousness disturbance, and gasping during the early stage; elevated C-reactive protein, procalcitonin and LDH levels; and non-improvement of disease condition or relapse following an initial response when antiviral treatment was administered. Once the presence of bacterial infection has been confirmed, empiric antibiotic therapy should be initiated as soon as possible.

## Data Availability

All data generated during this study are included in this published article.

## Abbreviations

PICU: Paediatric Intensive Care Unit
LDH: Lactate Dehydrogenase
